# Pregnancy in the Shadow of Covid‐19: A Qualitative Study

**DOI:** 10.1111/hex.70558

**Published:** 2026-01-05

**Authors:** Giti Ozgoli, Sedigheh Sedigh Mobarakabadi

**Affiliations:** ^1^ Department of Midwifery and Reproductive Health, Midwifery and Reproductive Health Research Center, School of Nursing and Midwifery Shahid Beheshti University of Medical Sciences Tehran Iran

**Keywords:** Covid‐19, experience, Iran, pandemics, postpartum period, pregnant mothers, qualitative research

## Abstract

**Introduction:**

Pregnancy is a unique time that can be affected by environmental events. This study was conducted to explain mothers' understanding of pregnancy during the Covid‐19 pandemic.

**Methods:**

This study was conducted with a qualitative content analysis approach. Data were obtained through in‐depth virtual semi‐structured interviews with 16 pregnant and postpartum mothers before access to the Covid‐19 vaccine in Iran, between January and May 2021. Participants were selected from an online announcement in Tehran, Iran. Data were analyzed by conventional qualitative content analysis introduced by Graneheim and Lundman (2004).

**Results:**

Data analysis showed that mothers who became pregnant during the outbreak of Covid‐19, despite the lack of access to the vaccine, fulfilled their previous decision to get pregnant. They had motivations for pregnancy and made a conscious decision for this purpose. Prenatal care during the Covid‐19 pandemic has had unique features. Prenatal care was a necessity; mothers adjusted themselves to prenatal care. The continuous midwifery model of care using telehealth helped pregnant women overcome the difficulties of the Covid‐19 pandemic. Covid‐19 made a special experience for expectant mothers, which included everything from disturbance of mental condition to adopting solutions.

**Conclusion:**

Pregnancy and prenatal care during the Covid‐19 pandemic were a unique experience. Psychological disturbances, the impact of the pandemic on the experience of pregnancy, and the effectiveness of prenatal care under the conditions governing the pandemic were some of the features of women's experience. In future possible pandemics, the experiences gained from the Covid‐19 pandemic, such as telehealth and continued care, could be used.

**Patient or Public Contribution:**

This study was informed by the need to understand the firsthand experiences of pregnant and postpartum mothers during a public health crisis. While patients or the public were not involved in the initial design of this study, their central and invaluable contribution lies in their participation as the key study subjects. The research is fundamentally built upon their lived experiences, which were shared through in‐depth interviews. The analysis and interpretation of the data are directly derived from their narratives, ensuring that the findings are grounded in patient perspectives. Participants were also invited to review initial transcripts and coded data to verify accuracy and resonance with their experiences, enhancing the credibility and transferability of the results.

## 1 Introduction

Pregnancy is a predictable, uniform psychological process. The way people handle pregnancy affects postnatal consistency [1]. The experience of pregnancy and maternal care was significantly affected by the pandemic of Covid‐19 [2, 3]. The Covid‐19 pandemic has significantly impacted many countries since the beginning of 2020 [4]. The pandemic brought unprecedented disruptions and placed a huge burden on health care systems around the world. To contain the spread of the disease, governments around the world instituted a range of public health measures including travel restrictions, forced quarantine, local or national quarantines, and social distancing [5]. Based on observations of previous viruses, experts have identified pregnant women as vulnerable and advised them to take further precautions [6]. Many hospitals limited the number of patients permitted to attend prenatal visits, during birth and postpartum visits [7]. In Iran, to protect the pregnant women along with routine recommendations and to increase the interval of pregnancy visits in low‐risk pregnancies, it was suggested. The number of pregnant women referred to the Para‐clinic assessment was limited [8].

Women who were pregnant or delivered at an early stage of the Covid‐19 pandemic, along with other potential physical and mental illnesses that may occur during pregnancy, faced significant uncertainty. The general population showed a threefold increase in emotional disorders such as depression during the Covid‐19 pandemic [9]. In Iran, anxiety was reported at 57.9% and depression was reported at 47.9% during the outbreak of Covid‐19 [10]. The unfortunate news regarding deaths, physical, mental and emotional exhaustion, the deaths of medical staff, a shortage of health facilities and secondary complications from Covid‐19 infection were among the cases that created confusion and anxiety among pregnant women in the pandemic crisis [11, 12]. So that pregnant mothers were under severe stress, their routine and peace of mind changed and faced new challenges due to the pandemic [2]. During this period, pregnant mothers reported a spectrum of experiences, on one side of which was the fear of being infected with the virus, the feeling of isolation and lack of support, the inability to share experiences, the disruption of prenatal care, and on the other side, the experience of a quiet period that provided an opportunity to get away from the preoccupations before the Covid‐19 pandemic [13].

Iran is a densely populated country [14] with about one million women births every year [15]. In Iran, prenatal care is provided in many public and private health centers. Care in the public sector is provided by healthcare centers. In addition, the private sector plays a large role in providing care. Also, private sector has almost a large department of laboratories and diagnostic facilities [16]. No doubt, at the time of the outbreak of Covid‐19, the multiple‐system surveillance had its own unique features.

Based on these, we had decided to conduct a qualitative study aimed at understanding women's experience of pregnancy during the pandemic of Covid‐19. This approach enables researchers to achieve a broad description of the phenomenon under study [17]. Knowledge of previous outbreaks [18, 19, 20] is helpful in the face of the Covid‐19 pandemic. Despite the diminishing of the Covid‐19 pandemic, the spread of knowledge during this period can enhance our preparedness in the face of possible future outbreaks.

## 2 Methods

### 2.1 Approach

We used qualitative content analysis approach to achieve the research goal. This research method enabled researchers to immerse themselves in the research data and achieve a new vision and a broad description of the phenomenon under study [17].

### 2.2 Sampling

Participants were selected from online announcement in Tehran, Iran. The characteristics of entering the study included: being Iranian, ability to read and write, ability to speak Persian, age between 19 and 35 years, being pregnant, or giving birth after the outbreak of the Covid‐19 virus, singleton pregnancy, uncomplicated pregnancy (bleeding, cervical insufficiency, preeclampsia, etc.), the absence of medical complication (high blood pressure, diabetes, etc.), the absence of obvious infection with the Covid‐19 virus. Thirteen postpartum mothers and 3 pregnant women participated in this study. Mothers were pregnant at the start of the Covid‐19 outbreak or became pregnant during the first year of the Covid‐19 outbreak before the start of Covid‐19 vaccination. The mothers were selected by using targeted sampling with maximum variation regarding demographic and reproductive characteristics to collect a wide range of data. Among the individuals selected to participate in this study, 1 mother did not attend due to time limit.

### 2.3 Data Collection

Data were collected through a virtual semi‐structured in‐depth interview between January and May 2021. Fifteen interviews via WhatsApp messenger, and one interview was conducted by phone. The duration of each interview was between 30 and 85 min. The interview started with an open question: ‘Can you explain how your pregnancy coincided with the covid‐19 pandemic? Tell me what happened inside you?’, and it continued with ‘what happened and what did you do?’, and more querying questions were used with the participants' answers, like: ‘Can you tell us more about this?’ All interviews were recorded, typed word by word, and Data analysis started simultaneously with data collection. Data collection continued until no new information was received from the interviews that is to say saturation of information [21].

### 2.4 Ethical Considerations

This research has been approved by the ethics committee of Shahid Beheshti University of Medical Sciences (Ethical approval code: IR.SBMU.RETECH.REC.1399.861). Also, informed electronic consent was sent to the participants before the time of the interview and after getting consent, the time of the interview was coordinated. All participants were assured that they could not continue to participate in the study anywhere they chose. To maintain confidentiality, all interviews were recorded via code and without the names of the participants.

### 2.5 Data Analysis

The data was collected and analyzed using conventional qualitative content analysis method introduced by Graneheim and Lundman [22]. Main theme and categories were extracted. The entire interview was considered as unit of analysis. The whole interview was studied over and over again until the researcher immersed in the data and was able to extract the meaning units. Then the meaning units were summarised, and encryption was performed in the next stage. The codes were categorized based on semantic similarity and the categories were formed, below the classes with the same meaning, and the final theme was formed through the meanings behind the floors that connected them [22].

MAXqda (portable 2007, udo Kuckartz Berlin/Germany) was used for data analysis. To promote the trustworthiness of the research, interviews were conducted by two qualitative investigators in order to maintain the credibility. All analyses for similarity and negative points were evaluated by two qualitative researchers. Participants were given some of the text of the interview with the code to be verified, or reevaluated if a bug exists, or added to the list if more information came up. Two mothers who did not participated in the study, were asked to check the transcripts and codes to improve its transferability [23].

### 2.6 Results

This qualitative content analysis was conducted on 16 pregnant and postpartum mothers with an age range of 19 to 35 years. The demographic and reproductive characteristics of participants are shown in Table 1. Participants were identified with P and the adjacent number in the text. Quotes are showed as italics. From the data analysis of this study, three main themes were extracted, namely, ‘From pregnancy planning to implement’, ‘Prenatal Care in the Shadow of Covid‐19’, and ‘Covid‐19 Association with Pregnancy’ (Table 2). These themes are described in the next section.

**Table 1:** Characteristics of participants.

Parity	[*n* (%)]
Primiparous	10 (62.50)
Multiparous	6 (37.50)
Mother status	
Pregnant	3 (18.75)
Parturient (NVD)	6 (37.50)
Parturient (CS)	6 (37.50)
Parturient (VBAC)	1 (6.25)
Pregnancy status at the onset of the COVID‐19 outbreak	
Pregnant [gestational age (week)]	10 (62.50)
Not pregnant	6 (37.50)
Gestational age during the outbreak of COVID‐19 [gestational age (week)]	
1–13	5 (50.00)
14–26	5 (50.00)
Educational level	
Secondary school	2 (12.50)
High school	5 (31.25)
University	6 (37.50)
Postgraduate	3 (18.75)
Occupational status	
Housewife	10 (62.50)
Employed	6 (37.50)
Prenatal Health Provider	
Obstetrician	5 (31.25)
Midwife (In private office)	4 (25.00)
Midwife (in Health care center)	2 (12.50)
Obstetrician & Midwife	3 (18.75)
Obstetrician & health care center	2 (12.50)

Abbreviations: CS, Cesarean Section; NVD, Natural Vaginal Delivery; VBAC, Vaginal Birth After Cesarean Delivery.

**Table 2:** Theme, categories, sub‐categories and codes.

Them	Categories	Subcategories
1‐ From Pregnancy Planning to Implementation	1‐1‐ Pregnancy Decision	Motivations for pregnancy (irreversible fertility, pre‐existing plans); Informed decision‐making amidst risk.
	2‐1‐ Challenges of the Decision	Worry for personal/fetal health and existing children; Doubt about the decision's wisdom.
	3‐1‐ Strategies for Managing Risk and Anxiety	Rationalizing risk; Adherence to health protocols; Pre‐pregnancy health optimization; Self‐quarantine.
2‐ Prenatal Care in the Shadow of COVID‐19	1‐2‐ Reconciling Necessity with Fear: Self‐Protection as a Strategy	Prioritization of routine prenatal care; Adoption of protective measures (private transport, hygiene).
	2‐2‐ Divergent Experiences Across Care Models	Midwifery Model: Valued for continuity, support, and telehealth adaptation.
		Medical/Healthcare Models: Varied experiences; discontinuation due to inflexibility, crowding, or lack of safety; continuation where communication and flexibility were present.
	2.3. Para‐clinical Investigations: A Source of Significant Stress	Anxiety about crowded, high‐risk environments; Strategies for coping (selecting private centers, limiting tests).
3. Pregnancy during COVID‐19: Navigating Fear, Loss, and Isolation	3.1. The Dual Burden of Vigilance and Isolation	Extreme self‐protection measures; Strained family relations due to non‐compliance; Profound loneliness and loss of anticipated pregnancy joy.
	3.2. Direct Encounters with COVID‐19 and Trauma	Distress from family infections; Traumatic bereavement and impeded mourning; Personal COVID‐19 symptoms; Temporary cessation of prenatal care following grief.
	3.3. Coping and Adaptation	Use of virtual spaces for connection and support; Reliance on immediate family; Engagement in spiritual practices.

#### 2.6.1 From Pregnancy Planning to Implement

The first theme resulting from this analysis was the decision to perform pregnancy, which consisted of three categories as follows.

##### 2.6.1.1 Decision to Pregnancy

This class consisted of two subcategories: the motivations to decide about pregnancy and make informed decisions. The uncertainty about the end of the pandemic, the reluctance to lose the opportunity to conceive, the irreversibility of reproductive opportunity, the loneliness of the first child, the unwillingness to give up the future of fertility due to fear of Covid‐19, the decision to exercise a prior fertility planning motivated the mothers to make their decision to have pregnancy despite all the concerns during the Covid‐19 outbreak.

There were no plans or forecasts to end the Covid‐19. But the fertility opportunity, if it was lost, would have been irreversible.

(P14)

However, the decision to be pregnant was informed that many mothers were aware of risks associated with pregnancy, instability of the conditions of Covid‐19‐related infection in the community, general lack of access to a Covid‐19 vaccine, yet pregnancy was a major decision to take despite awareness of the risks.

My decision was not based on the lack of awareness or the consolations that the coronavirus does not pass on to the fetus.

(P16)

##### 2.6.1.2 Challenges

The decision to get pregnant during this period created challenges for the mother. This class consists of two subclasses of worry and doubt. Worrying about her own health and the health of the fetus and the ultimate consequences of the decision created mental conflicts for the mother.

I was worried, if something happens to me, my older child will be motherless.

(P13)

After making the decision to get pregnant, the mothers faced doubts about whether they would be able to handle the pregnancy in the conditions of the Covid‐19 pandemic. The mother had doubts about the correctness and wisdom of such a decision.

I don't know if I made the right decision or not. (P5)

##### 2.6.1.3 Strategies

They developed strategies to handle these emotions, attempted to compare pregnancy during Covid‐19 outbreak with other life hazards, and to overcome concerns. They also have tried to stay safe, adhering more to the health protocols. In this regard, they attempted to ensure their health prior to pregnancy, control other health conditions and limit the risk that they can control. Some of the mothers step forward and try to stop activity outside the home and quarantine themselves at home.

I decided to prepare myself for pregnancy, I started taking folic acid, I took tests to make sure of my health, I tried to avoid the risks that I could and could at least start the pregnancy with preparation so that there is less risk, threaten me.

(P17)

#### 2.6.2 Prenatal Care in the Shadow of Covid‐19

Prenatal care during the COVID pandemic has had unique features. This theme consists of five categories as follows.

##### 2.6.2.1 Prenatal Care From Necessity to Difficulty

This category consists of two sub‐categories prenatal care is a necessity, self‐protection for immunity from the virus in prenatal care.

Even though the beginning of the pandemic was associated with many concerns, mothers understood routine care during pregnancy as a necessity that they could not give up even in such unique circumstances. Mothers were trying to perform all the routine care in person despite the pandemic, and they probably thought that they should pay more attention to prenatal care during this period. In their view, prenatal care had more priority than concerns caused by virus transmission.

I've gone to all my care, maybe more.

(P1)

Self‐protection before, during and after prenatal care was a strategy used by mothers to protect themselves and at the same time maintain routine prenatal care. Mothers tried not to use public transportation to go to the place of care. They should come to the place of visit following the health protocols, observe the social distance at the place of visit and perform the health protocols after the end of the care.

I would only go with our own car. Before that I would go there by bus or taxi.

(P2)

##### 2.6.2.2 Continuing Midwifery Model of Care

This category consisted of three subcategories: ‘Transition from the difficulties of Covid‐19 in pregnancy with the help of continuous midwifery care’, ‘Continuity of midwifery care during the outbreak of Covid‐19’, ‘Adjustment of pregnancy care with the help of telehealth’.

During this period, mothers considered midwifery care to be a useful care; they believed that they did not feel much tension in this care, this type of care facilitated the transition from the difficult period of the outbreak of Covid‐19. This care was satisfactory for mothers, midwives were considered to be a good person for care. They believed that a proper interaction was established with them during care. This type of care has helped to achieve natural childbirth in mothers during the critical conditions of the outbreak of Covid‐19.

I was really satisfied with the (midwife) I chose, the peace she gave me was so much. If it wasn't for the her help, I couldn't give birth naturally

(P3)

Care that had begun before the coronavirus outbreak continued, mothers had regular access to midwifery care and were able to receive full midwife support throughout their pregnancy. During this period, midwife attempted to provide regular access to pregnant mothers and reduce unnecessary face‐to‐face visits, while referring high‐risk mothers to higher‐level centers.

What made it possible for midwives to continue providing midwifery care and increasing support during the virus outbreak was the optimal use of communication tools and social media. In this way, the midwife managed to adjust the care during the virus outbreak. Telephone counselling, online counselling, providing educational books and CDs, holding live trainings on social media, even holding online childbirth preparation classes, were some of the solutions to continue the support.

Whenever there was a problem, we asked them at WhatsApp.

(P2)

##### 2.6.2.3 Medical Model of Care

This class consisted of two sub‐classes with the titles ‘abandoning medical care’ and ‘continuing medical care’. This model of care was one of the conventional types of prenatal care among the population from which the participants were selected. During the outbreak of Covid‐19, some pregnant mothers tried to change this care. The impossibility of continuous care despite the outbreak of the virus, unnecessary frequent referrals to high‐risk centers in terms of the virus, crowding in the place of care, not feeling relaxed, feeling of lack of attention to the mother's health, lack of respect for privacy, and visits mothers with covid‐19, was one of the reasons that led low‐risk mothers to change their type of care.

It was not possible to contact my doctor by phone.

(P5)

But there were mothers who remained in medical model of care. Lack of congestion in the maternity care site due to proper timing for the presence of mothers, possibility of making phone calls, favourable interaction with mother and creating peace in the mother were some of the cases which encouraged mothers to stay in this type of care during the virus outbreak.

I had both the doctor's and the secretary's number. Saying that if it is an acute problem, call the doctor.

(P9)

##### 2.6.2.4 Healthcare Model

This category was made up of two subcategories under the headings ‘Confronting the Challenge in Health Care’ and ‘Ensuring Continuity of Health Care in Special Conditions’. Another type of prenatal care provided by the health center was provided in the population under study. During the outbreak of Covid‐19, although a number of pregnant mothers received care in this way and continued until the end of pregnancy, some mothers refused to continue prenatal care at the healthcare center for the following reasons: Congestion at the place of care, people with symptoms related to Covid‐19 go to health centers, failure to observe social distance, dissatisfaction with care, impossibility of face‐to‐face communication

Healthcare center was overcrowded and didn't adhere to the health protocol. They did not answer online or on the phone.

(P9)

##### 2.6.2.5 Para‐Clinical Investigations, From Stress to Risk Adjustment

This category was composed of three subcategories: ‘Stressful Para‐clinical investigation’, ‘Risk Adjustment by the Para‐clinical Sectors for Mother's Protection’ and ‘Mother's Solutions for Self‐Protection’.

Para‐clinical studies during the Covid‐19 outbreak were highly stressful for mothers who considered going out of their home or going into the environment risky in conducting these studies. They were thus placed in a very busy environment, where social distancing was not possible. Sometimes there were no primary health facilities in the government sector. Mothers, after settling in these environments, would find themselves a potential risk to the family. Private sectors were more discreet and had better access to health protocols, but mothers faced higher costs.

Para‐clinical sectors also understood this concern of mothers and tried to reduce such anxiety by health protocols, keeping distance between clients, limiting the presence of companions, using disposable devices, frequent disinfection of surfaces and equipment, limiting the entry of people without masks and reassuring of mother.

Meanwhile, in order to overcome the stress and protect their self and the fetus, the mother took some solutions, including the mother tried to choose a clean and quiet center that was mostly in the private sector, in order to feel safe. They also tried to follow the health protocols during and after visiting such centers and only carry out necessary examinations.

As soon as I went to those environments, I was stressed. Wherever I went, they tried their best to follow all the protocols.

(P4)

#### 2.6.3 Covid‐19 Association With Pregnancy

The third theme resulting from this analysis included three categories as follows.

##### 2.6.3.1 Strategies to Prevent Virus Infection and Challenges Arising From It

The class consisted of six sub‐categories, namely ‘mother solutions for self‐protection’, ‘others' psycho‐social supports’, ‘difficulty in continuing the implementation of health protocols with the development of a pregnancy’, ‘facing mental and physical challenges following the application of health protocols’, ‘coping with emotional difficulties’ and ‘facing communication challenges’.

During this sensitive time, mothers struggled to protect themselves and their fetus more. For this purpose, they committed their self to abide by health protocols, including the use of face masks, the use of hand washing, observing social distancing, restrictions on outdoor activity and entertainment, stay in quarantine, communication only with close relatives who followed health protocols.

I always wore two masks wherever I wanted to go.

(P10)

In addition to the mother, the family tried to support the pregnant mother during this period by preventive measures. Husband and close relatives who were in contact with pregnant mother tried to follow health protocols to reduce the chances of the virus spreading to the mother. They were attempting to support the mother's psychological well‐being and thus protect the mother's mental health during this period.

My husband was very attentive to me. He took great care.

(P4)

Compliance with the health protocols was not easy. With the growing age of pregnancy, compliance with the stricter protocols was becoming apparent. The difficulty of breathing at the end of the pregnancy made the masks more difficult to tolerate. The warming of the weather made the situation even more difficult. The incidence of obsessive‐compulsive care in a person or close relatives to protect the mother was some of the challenges of mothers.

My husband became obsessed because of me.

(P4)

Mothers were also concerned about their ongoing relationship with relatives who didn't follow health protocols, but they still insisted on continuing to visit the mother. Therefore, they limited the relations with this group, which created a double challenge for mothers.

My husband's family didn't follow health protocols. I said that I can't meet them because I'm pregnant. There was a lot of pressure. My husband would have been unhappy. We've been challenging. We've had an argument.

(P2)

##### 2.6.3.2 Exposure to Covid‐19 From Infection **of the Surrounding People** to Pregnant Mother

This category included three sub‐categories with the titles of ‘Family members being infected with Covid‐19’, ‘Death of relatives due to Covid‐19’, ‘Mother's exposure to Covid‐19 symptoms during pregnancy’.

Family members were infected with Covid‐19, and as a result, the mother did not meet with the family members, and sometimes she left her place of residence and went to a safe place for the pregnant mother, all of which caused her to feel uncomfortable. Mother, following the illness of relatives, she wishes to be reunited in the future. Among them, there were mothers who did not care about the possibility of contracting Covid‐19 and tried to support the infected person even from a distance.

We lived with my husband's family in our building until the husband's family got infected with Corona virus. My father‐in‐law said that if you stay here, you will surely get Corona virus and this may endanger your life and the life of your baby, so leave here as soon as possible.

(P7)

Faced with the death of relatives, friends and family members due to Covid‐19 infection, it was a difficult event for mothers and some mothers faced a feeling of shock. At the same time, the restrictions caused by the spread of Covid‐19 led to the impossibility of mourning, which also made it more difficult for the mother to bear the death of her relatives. In the meantime, some mothers faced other problems, such as the quarantine of their spouses due to contact with a person who died of Covid‐19, which increased the emotional pressure caused by this incident for the mother. Some mothers lost their desire to receive prenatal care as a result of these emotional pressures. This caused the mother to face complications during pregnancy. Faced with the news of the death of other pregnant mothers, mothers faced depression and fear of death because they felt this danger for themselves.

My father‐in‐law died due to covid‐19. I was crying all the time. My husband did not even come to see me. He couldn't even hug me when I needed him. I did not participate in any mourning ceremony. I was bored. During that time, I did not go for prenatal care at all.

(P8)

Among these, there have been situations where the mother faced the symptoms of covid‐19 during pregnancy. In this situation, they tried to protect others. Mothers felt worried, worried about their health and the health of the fetus, and every day they expected the symptoms to intensify

I had lost my sense of smell for a week. I was very afraid. Every day I waited for more signs.

(P1)

##### 2.6.3.3 Disturbance of the Mother's Mental Condition and Trying to Adapt

This category included five subcategories with the titles of ‘mood changes’, ‘mother's feeling towards pregnancy’, ‘feelings caused by quarantine’, ‘pregnancy accompanying stress’ and ‘solutions to cope with mood changes’.

During this period, mothers experienced state of boredom, mental conflict, loneliness, sadness, depression, and loss of spirit, fear of death, concerns and uncertainty that were extremely difficult for them. Pregnant women described the experience of excessive crying in pregnancy and exposure to disturbed sleep during night insomnia. Mothers raised many concerns stemming from the spread of the virus.

I was obsessed. I used to dream a lot. I thought at night. I cried for nothing.

(P1)

The spread of the virus affected mothers' feelings about pregnancy. Mothers felt that the pregnancy which was supposed to be a sweet baby passed through difficult times and that the sweetness of the pregnancy became difficult to cope with the virus and the unpleasant news associated with it. This experience upset the mother.

Corona didn't let me feel the pregnancy confectionery.

(P12)

Quarantine during Covid‐19 periods could produce a sense of depression, having to stay at home, facing undesired emotion, not being satisfied with the need for entertainment and dealing with stress if left home.

You need more fun in pregnancy. This requirement could not be met.

(P16)

During Covid‐19, pregnant women have always faced stress, a stress that was at first very severe due to the fact that Covid‐19 was not known, but over time more information about the virus and compliance with health protocols was discontinued to some extent. Mothers found they were naturally experiencing stress during pregnancy which increased the risk of Covid‐19.

The risk of developing the disease in pregnancy, maternal and fetal outcomes, the presence of the death of those around the virus and financial pressures as a result of the spread of the virus in the community, among the causes of stress during pregnancy in mothers were studied.

During pregnancy, the mother has some stress, some symptoms that she does not know if it is normal or not, and the stress of childbirth and many other things, the stress of Corona was added to all these stresses.

(P14)

Mothers struggled to overcome the psychological challenges caused by Covid‐19 to find a way to communicate with the outside world via virtual space. The smartphone went for fun and communication with the outside world. They used TV for entertainment. They tried to overcome the challenge of loneliness and isolation by meeting their parents and important people around them who followed health protocols. Prayer and worship, morale‐strengthening lectures, presence of the first child at home, attempting to entertain, support with midwives were among other ways to help mothers overcome the psychological challenges associated with Covid‐19.

The virtual space was very much able to tolerate the quarantine in this period.

(p13)

## 3 Discussion

This study was conducted to assess the experience of mothers during the Covid‐19 outbreak with the help of qualitative conventional content analysis. Data analysis showed that some mothers made their previous decision on fertility when the Covid‐19 outbreak occurred. Prenatal care influenced by Covid‐19 took on special features, and Covid‐19 provided specific experience to mothers during this period.

The limited time of fertility and uncertainty with the end of the Covid‐19 virus outbreak prompted people to enforce a decision to occur before the outbreak of Covid‐19. The decision was made knowing about the mother's danger and the mothers sought to use the available means to reduce the risk. However, the concern, along with the realization of this decision, was not eliminated. In a study by Flynn et al. in the UK, 92% of women were planning to be pregnant at the time of the Covid‐19 outbreak, but more than half of the participants reported that Covid‐19 had influenced their schedules, with 72% of people purposely delaying pregnancy. Concerns were mainly about changes in prenatal care as well as the fear of adverse effects of the virus on mother and baby [24]. In our study, contrary to the above study, pregnant mother were studied. Therefore, we do not know about the understanding of people who decided to get pregnant and do not implement their decision due to the Covid‐19 pandemic.

Prenatal care during the Covid‐19 pandemic has had unique features. While some mothers prioritized prenatal care over virus transmission concerns, others began to adjust their care. The type of care received also helped to adjust the care. So that in medical model of care during pregnancy and health care model, some mothers refused to continue these two types of conventional care in the study environment due to facing challenges. In the Mortazavi and Ghardashi study in Iran, efforts to prevent people from visiting health centers, closure of some clinics, cancellation of appointments, closure of some specialised private practices, increased waiting times for visits to hospitals, and congestion of visitors to ultrasound clinics and other types of clinics were identified as the challenges experienced by pregnant women to receive care at the beginning of Covid‐19 pandemic [2]. Erchick et al. reported in a study in the United States that Covid‐19 pandemic changed prenatal care plans for most pregnant women in 2020. The researchers reported reduced visits and increased delivery in the home and delivery caesarean section [25]. Groulx et al. found in a study in Canada that cancellation of prenatal care appointments and changes in delivery schedule during the Covid‐19 outbreak were more likely to be associated with clinical experience of increased depression, anxiety, or pregnancy‐related anxiety symptoms [26].

In our study, mothers who were in regular midwifery care survived this type of care upon the onset of the Covid‐19 pandemic and through telehealth, with the help of midwife's potential cyberspace, were able to satisfy many of their care needs and avoid unnecessary face‐to‐face visits. Fryer et al. considered telehealth critical to ensuring safe and effective delivery of midwifery care due to the Covid‐19 pandemic. It has also suggested that planning, processes and thoughtful evaluation are necessary to ensure that telehealth sustains after the outbreak of Covid‐19 [27]. In the Brasdfield study, et al. of the 620 midwives participating in the study reported 56.5% to telehealth in the course of the Covid‐19 pandemic [28].

Self‐protection before, during and after prenatal care was a strategy used by mothers to protect themselves and at the same time maintain routine care. Of course, at the beginning of the pandemic, there was a noticeable lack of health products such as gel and health masks in hospitals, health centers and pharmacies [2], which probably created restrictions for some mothers. Also, mothers tried not to use public transportation to go to the place of care. Because the risk of human infection due to the length of the exposure time window, transmission routes and structural features could lead to rapid spread of infection [29].

Virus prevention strategies created challenges for mothers. Also, encountering Covid‐19 during pregnancy resulted in a unique experience for mothers, which disturbed the mental condition of the mother during this period, which, of course, resulted in trying to adapt to these conditions. Mortazavi and Ghardashi found that pregnant women experienced severe difficulties and stress in their daily lives during quarantine in Iran. Stress, fear, worry and anxiety, feelings of depression and loneliness were common among pregnant women during the pandemic. These researchers found that, compared to other groups, pregnant women have other concerns besides their health. They were concerned about the health of the fetus and having a healthy delivery [2]. In the study of Puertas‐Gonzalez et al. phobic anxiety, perceived stress, depression and insomnia were increased in pregnant women during the period of Covid‐19 pandemics [30]. Ahmad and Vismara reported a moderate to severe impact of the Covid‐19 outbreak on the mental health of pregnant women, mainly in the form of a significant increase in depression and anxiety symptoms [31]. In the meta‐analysis reported by Demissie and Bitew in pregnant and lactating women, the cumulative prevalence of anxiety was 33%, depression 27%, stress 56%, insomnia 33.53%, social dysfunction 24.3%. In fact, stress was the most common mental health problem in these population groups [32].

## 4 Conclusion

This study was one of the studies conducted on the experience of mothers in pregnancy during the pandemic of Covid‐19 before they had access to vaccinations. Among the limitations of this study were conducting interviews online and by phone, which limited the observation of the person's facial expressions. It is recommended to conduct in‐person interviews to test mothers' experience with the pandemic of Covid‐19 as the risk of virus spreads is minimized. This study sought to provide a broad description of maternal experience during the outbreak of Covid‐19, which may be helpful in similar cases in the future. Also, this study showed that continuous care, especially for crises such as the pandemic covid‐19, along with the use of telehealth, improves the continuity of prenatal care by the mother.

## Author Contributions

**Giti Ozgoli** and **Sedigheh Sedigh Mobarakabadi:** conceptualization, investigation, methodology, validation, formal analysis, software, data curation, supervision, project administration, writing – review and editing, writing – original draft, resources.

## Funding

The authors received no specific funding for this work.

## Ethics Statement

This research was approved by the ethics committee of Shahid Beheshti University of Medical Sciences (Ethical approval code: IR.SBMU.RETECH.REC.1399.861). Additionally, informed electronic consent was sent to the participants before the interview, and after providing consent, the interview time was coordinated. All participants were assured that they could not continue to participate in the study anywhere they chose. To maintain confidentiality, all interviews were recorded anonymously, using a code to identify participants.

## Conflicts of Interest

The authors declare no conflicts of interest.

## Data Availability

The data that support the findings of this study are available on request from the corresponding author. The data are not publicly available due to privacy or ethical restrictions.
